# Crystal structure of 3,5-di­methyl­pyridine *N*-oxide dihydrate

**DOI:** 10.1107/S205698901601687X

**Published:** 2016-11-01

**Authors:** Rosario Merino García, Francisco Javier Ríos-Merino, Sylvain Bernès, Yasmi Reyes-Ortega

**Affiliations:** a Centro de Química, Instituto de Ciencias, Benemérita Universidad Autónoma de Puebla, 72570 Puebla, Pue., Mexico; b Instituto de Física, Benemérita Universidad Autónoma de Puebla, Av. San Claudio y 18 Sur, 72570 Puebla, Pue., Mexico

**Keywords:** crystal structure, lutidine, dative bond, hydrate, ring motif

## Abstract

In the title compound, also known as 3,5-lutidine *N*-oxide dihydrate, C_7_H_9_NO·2H_2_O, the N—O bond is weakened due to the involvement of the O atom as an acceptor of hydrogen bonds from the two water mol­ecules of crystallization present in the asymmetric unit. Fused *R* ^3^_5_(10) ring motifs based on O—H⋯O hydrogen bonds form chains in the [010] direction, which are further connected by weak C—H⋯O inter­molecular contacts. As a result, the lutidine mol­ecules are stacked in an efficient manner, with π–π contacts characterized by a short separation of 3.569 (1) Å between the benzene rings.

## Abstract 2

In the title hydrate, water mol­ecules and *N*-oxide groups of the main mol­ecule form supra­molecular chains based on *R*(10) ring motifs.

## Chemical context   

Dimethyl-substituted pyridines, commonly known as lutidines, are useful small organic co-ligands for coordination chemistry, since the position of the two methyl groups on the ring modulates the nucleophilic character of the donor N atom (*e.g*. Xu *et al.*, 2010 ▸). Corresponding *N*-oxides, which are much less basic, are readily accessible, and have different applications. For example, 3,5-lutidine *N*-oxide has been used as an additive in radical polymerization of *N*-alkyl­acryl­amides, inducing a significant level of isotactic polymerization (Hirano *et al.*, 2009 ▸).
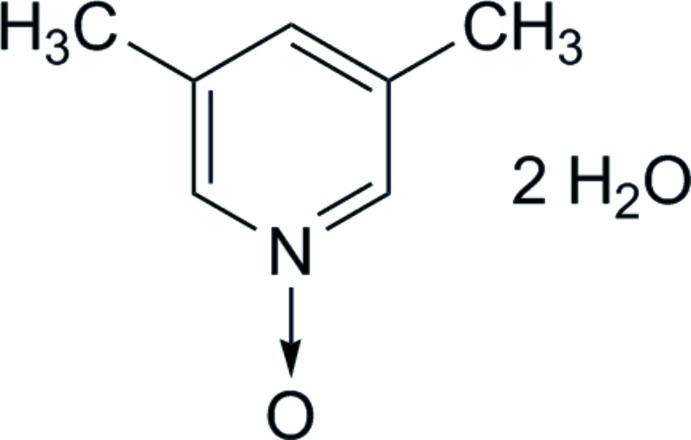


The *N*-oxide formation can also be used to temporarily activate the pyridine or lutidine ring, to both nucleophilic and electrophilic attack. For example, pyridine *N*-oxide readily undergoes nucleophilic addition followed by elimination, providing useful synthesis of 2-substituted pyridines. While working on the synthesis of 2-amino-pyridine-3,5-di­carb­oxy­lic acid starting from 3,5-lutidine, we crystallized the title compound as an inter­mediate, and determined its crystal structure. As expected, the mol­ecular structure shows no unexpected features, while the arrangement of water mol­ecules in the crystal is more inter­esting, showing why the crystallization of the dihydrate is favoured.

## Structural commentary   

**Figure 1:**
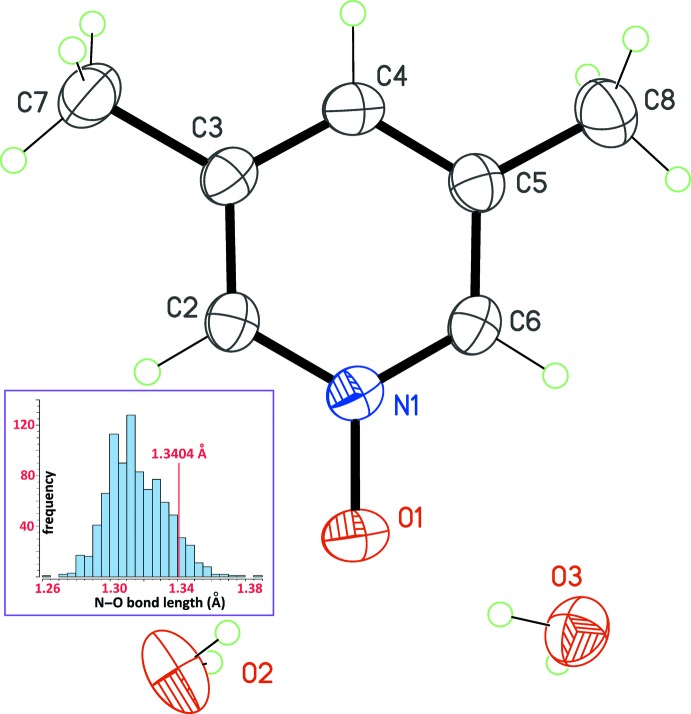
The structure of the title compound, with displacement ellipsoids for non-H atoms at the 30% probability level. Only one orientation for methyl groups C7 and C8 is retained. The inset is the distribution for the N—O bond lengths of pyridine *N*-oxide derivatives in the organic subset of the CSD (updated May 2016; Groom *et al.*, 2016 ▸). 673 hits were retrieved for which the O atom gives a single bond, affording 904 raw data. Eight outliers were omitted, and the 896 used data gave a mean value for the N—O bond length of 1.316 Å. The red line locates the bond length in the title compound.

The 3,5-lutidine *N*-oxide mol­ecule potentially displays *C*_2*v*_ mol­ecular symmetry. However, the mol­ecule is found in a general position, perhaps because the rotational disorder affecting the methyl groups breaks this latent symmetry. The asymmetric unit is completed by two water mol­ecules of crystallization in the close vicinity of the N—O bond (Fig. 1 ▸).

The bond length for the *N*-oxide group, 1.3404 (14) Å, is comparable with those found in many other pyridine *N*-oxides: in the organic subset of the Cambridge Structural Database (CSD, updated May 2016; Groom *et al.*, 2016 ▸), this bond length presents a normal distribution around the mean value of 1.316 Å (Fig. 1 ▸, inset). In the title hydrate, the N—O bond length falls in the upper qu­antile of this statistical distribution, reflecting a slight weakening of the bond.

The N—O bond has been described in great details in a recent article (Łukomska *et al.*, 2015 ▸), both from the theoret­ical and statistical points of view. It has been shown that for pyridine *N*-oxide and related aromatic oxides, there is a significant stabilizing π-type O→N back-donation, reflected in a calculated bond order higher than 1 and a number of electron lone pairs on the O atom lower than 3. For the title hydrate, the weakly electron-donating groups in *meta* positions on the pyridine should have negligible influence on the N—O bond. In contrast, the strong Lewis basicity of the *N*-oxide should favour hydrogen bonding with the water mol­ecules. The charge is transferred from the O atom to the water mol­ecules (Lewis acid) at the expense of O→N back-donation, leading to N—O bond weakening and bond-length elongation, as observed. This behaviour is consistent with the IR data: the stretching vibration ν_N—O_ is found at 1307 cm^−1^ for our compound, shifted to lower wavenumbers compared to non-inter­acting pyridine *N*-oxide in the gas phase (1320 cm^−1^, as computed by Łukomska *et al.*, 2015 ▸). Hence, both the crystallographic and spectroscopic features observed for the N—O bond in the title hydrate suggest that this bond is essentially similar to that of pyridine *N*-oxide, and should be considered as an actual non-polar dative bond N→O, rather than a polar covalent bond N^+^—O^−^.

## Supra­molecular features   

**Figure 2:**
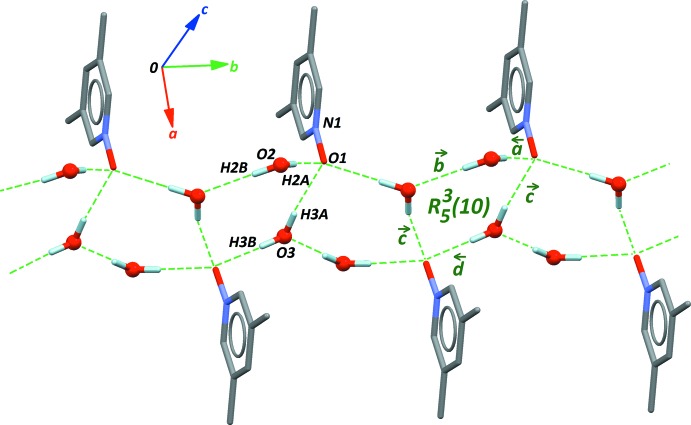
The main supra­molecular framework in the crystal structure. Hydrogen bonds *a*–*d* are described in Table 1 ▸. The pathway for ring motif *R*(10) starts from O1 and is oriented counterclockwise.

The crystal structure is dominated by hydrogen bonds between the water mol­ecules and the N—O group. Four O—H⋯O contacts build 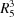
(10) ring motifs. This fourth level motif, with pattern **R**(<*a*>*b*>*c*<*d*>*c*), displays an envelope conformation, and is fused with the neighbouring *R* motif through the bond labelled *c* (Table 1 ▸, Fig. 2 ▸; ring starting from O1). As a consequence, rings of higher degree are formed, 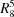
(16), 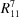
(22),⋯, *R* ^2*n*+1^_3*n*+2_(6*n* + 4), to give a one-dimensional supra­molecular network in the [010] direction (Fig. 2 ▸). From the four hydrogen bonds included in this motif, three are based on the N—O group as acceptor (bonds *a*, *c* and *d*, see Table 1 ▸), suggesting that the number of lone pairs on the O atom of the *N*-oxide group is close to 3. These hydrogen bonds have their O—H⋯O angles close to linearity, and should thus contribute to a large extent to the stabilization of the dihydrate.

**Figure 3:**
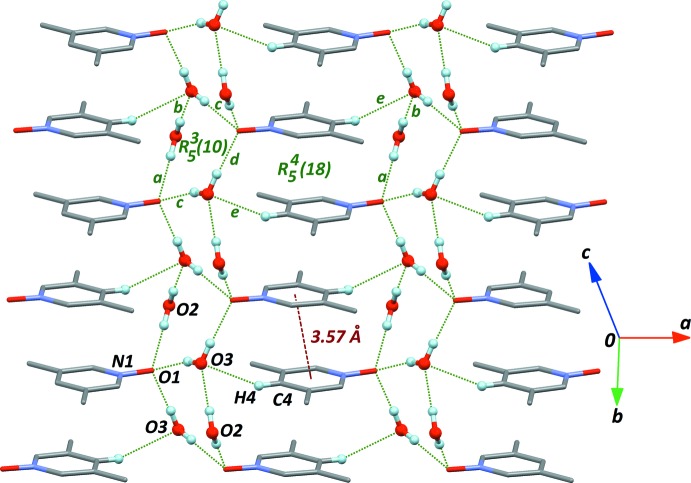
Stacking of aromatic rings in the crystal structure, *via* the secondary inter­molecular contact *e*, described in Table 1 ▸.

**Figure 4:**
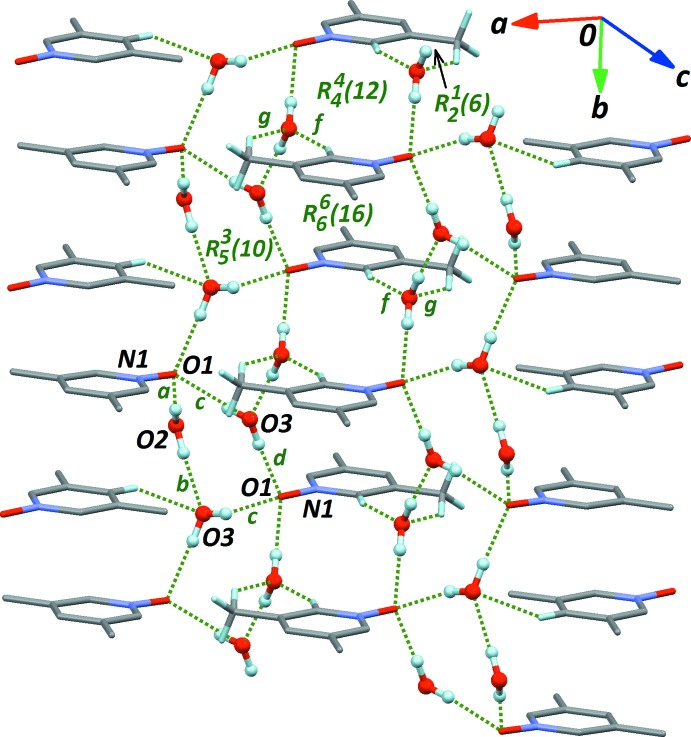
Participation of secondary inter­molecular contacts *f* and *g* (see Table 1 ▸) in the formation of ring motifs *R*(6), *R*(12) and *R*(16).

The supra­molecular structure is actually more complex if one considers secondary weak inter­actions between the [010] chains. The first contact, C4—H4⋯O3^ii^ (Table 1 ▸, entry *e*), connects two parallel chains and induces π–π inter­actions, characterized by a short contact distance between the benzene rings of 3.569 (1) Å. Inter­acting rings along the stack are almost parallel, the angle between neighbouring benzene rings being 2.13 (1)°. Stacked mol­ecules and water mol­ecules framework form 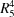
(18) rings (Fig. 3 ▸). Finally, two other weak C—H⋯O inter­actions with water mol­ecule O2 (Table 1 ▸, entries *f* and *g*) also connect the main one-dimensional framework (Fig. 4 ▸), forming a number of new *R* motifs in the crystal, with different sizes, *R*(6), *R*(12), and *R*(16). However, no π–π contacts are formed on the basis of these rings. The three C—H⋯O inter­actions *e*, *f* and *g* are of limited strength, although they probably do not occur by chance, and should then have some influence on the observed packing arrangement (Taylor, 2016 ▸).

## Database survey   

All lutidine isomers are commercially available, and are substances that are liquid at room temperature, with melting points ranging from 213 to 267 K. However, crystal structures for all the six possible isomers have been determined and reported in this journal, by the group headed by Andrew Bond at the University of Cambridge, UK. Crystals were obtained by *in situ* growth from the liquid, in glass capillary tubes, at a temperature just below the melting point of each isomer (Bond *et al.*, 2001 ▸; Bond & Davies, 2002*a* ▸,*b* ▸,*c* ▸,*d* ▸; Bond & Parsons, 2002 ▸). Moreover, lutidines appear frequently as solvents of crystallization (*e.g*. Xu *et al.*, 2005 ▸), as monodentate ligands (*e.g*. Wölper *et al.*, 2010 ▸), or as components of co-crystals (*e.g*. Schmidtmann & Wilson, 2008 ▸).

Regarding lutidine *N*-oxides, only two isomers have been described crystallographically. 2,6-Lutidine *N*-oxide monohydrate has a crystal structure featuring helicoidal one-dimensional supra­molecular chains formed through hydrogen bonds of moderate strength (Planas *et al.*, 2006 ▸). Other compounds with this isomer are essentially coordination compounds. 3,5-Lutidine *N*-oxide has been much less used; however, a recent study uses this oxide as a ligand for the synthesis of an Mn^III^–porphyrin complex (Pascual-Álvarez *et al.*, 2015 ▸).

## Synthesis and crystallization   

The title compound was obtained following the methodology reported for the synthesis of pyridine *N*-oxide (Ochiai, 1953 ▸). A mixture of glacial acetic acid (0.5 mol), 3,5-di­methyl­pyridine (0.051 mol) and hydrogen peroxide (35% solution, 8.5 ml) was heated at 353 K for 5 h, under constant stirring. The reaction was then cooled, and the excess of acetic acid distilled under reduced pressure. Water (10 ml) was added and the mixture was concentrated as far as possible. After dilution with water, the pH was adjusted to 10 with Na_2_CO_3_, and the solution was extracted with CHCl_3_ and dried over Na_2_SO_4_.

After filtration, the solvent was eliminated under reduced pressure, affording a very hygroscopic beige–white crystalline powder (70%). The same strong hygroscopic character was previously noted for pyridine *N*-oxide (Ülkü *et al.*, 1971 ▸; Patyk *et al.*, 2014 ▸). The powder was dissolved in diethyl ether and left to slowly evaporate at 277 K, to give clear colourless crystals (m.p. 310–311 K).

## Refinement   

Crystal data, data collection and structure refinement details are summarized in Table 2 ▸. Both methyl groups C7 and C8 are disordered by rotation about their C—C bonds. For each methyl, two groups of H atoms were first located in difference maps, and eventually restrained to ideal tetra­hedral CH_3_ groups, with occupancies for all H atoms fixed to ½. For water mol­ecules O2/O3, H atoms were found in difference maps and refined with free coordinates and *U*_iso_(H) = 1.5*U*_eq_(O2/O3).

## Supplementary Material

Crystal structure: contains datablock(s) I, global. DOI: 10.1107/S205698901601687X/hb7624sup1.cif

Structure factors: contains datablock(s) I. DOI: 10.1107/S205698901601687X/hb7624Isup2.hkl

Supporting information file. DOI: 10.1107/S205698901601687X/hb7624Isup3.cml

CCDC reference: 1510914

Additional supporting information: crystallographic information; 3D view; checkCIF report

## Figures and Tables

**Table 1:** Hydrogen-bond geometry (Å, °)

Entry	H bond	*D*—H	H⋯*A*	*D*⋯*A*	*D*—H⋯*A*
*a*	O2—H2*A*⋯O1	0.87 (3)	1.98 (3)	2.8489 (18)	179 (3)
*b*	O2—H2*B*⋯O3^i^	0.87 (3)	1.94 (3)	2.815 (2)	178 (3)
*c*	O3—H3*A*⋯O1	0.87 (3)	1.96 (3)	2.8053 (17)	162 (2)
*d*	O3—H3*B*⋯O1^i^	0.87 (3)	1.92 (3)	2.7875 (17)	176 (2)
*e*	C4—H4⋯O3^ii^	0.93	2.62	3.484 (2)	155
*f*	C2—H2⋯O2^iii^	0.93	2.36	3.246 (2)	158
*g*	C7—H7*D*⋯O2^iii^	0.96	2.65	3.453 (2)	141

Symmetry codes: (i) −*x* + 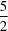, *y* − 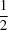, −*z* + 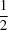; (ii) *x* − 1, *y*, *z*; (iii) −*x* + 2, −*y*, −*z* + 1.

**Table 2:** Experimental details

Crystal data	
Chemical formula	C_7_H_9_NO·2H_2_O
*M*_r_	159.18
Crystal system, space group	Monoclinic, *P*2_1_/*n*
Temperature (K)	296
*a*, *b*, *c* (Å)	8.7709 (12), 6.9476 (9), 14.5290 (17)
β (°)	90.966 (12)
*V* (Å^3^)	885.2 (2)
*Z*	4
Radiation type	Mo *K*α
μ (mm^−1^)	0.09
Crystal size (mm)	0.45 × 0.23 × 0.18

Data collection	
Diffractometer	Agilent Xcalibur Atlas Gemini
Absorption correction	Analytical (*CrysAlis PRO*; Agilent, 2013 ▸)
*T*_min_, *T*_max_	0.896, 0.952
No. of measured, independent and observed [*I* > 2σ(*I*)] reflections	15448, 2396, 1348
*R*_int_	0.038
(sin θ/λ)_max_ (Å^−1^)	0.700

Refinement	
*R*[*F* ^2^ > 2σ(*F* ^2^)], *wR*(*F* ^2^), *S*	0.047, 0.141, 1.02
No. of reflections	2396
No. of parameters	114
H-atom treatment	H atoms treated by a mixture of independent and constrained refinement
Δρ_max_, Δρ_min_ (e Å^−3^)	0.11, −0.15

Computer programs: *CrysAlis PRO* (Agilent, 2013 ▸), *SHELXT* (Sheldrick, 2015*a* ▸), *SHELXL2014* (Sheldrick, 2015*b* ▸), *XP* in *SHELXTL* (Sheldrick, 2008 ▸), *Mercury* (Macrae *et al.*, 2008 ▸) and *CIFTAB* (Sheldrick, 2008 ▸).

## supplementary crystallographic information

### Crystal data

**Table:**

C_7_H_9_NO·2H_2_O	*D*_x_ = 1.194 Mg m^−^^3^
*M**_r_* = 159.18	Melting point: 310 K
Monoclinic, *P*2_1_/*n*	Mo *K*α radiation, λ = 0.71073 Å
*a* = 8.7709 (12) Å	Cell parameters from 3054 reflections
*b* = 6.9476 (9) Å	θ = 4.0–25.6°
*c* = 14.5290 (17) Å	µ = 0.09 mm^−^^1^
β = 90.966 (12)°	*T* = 296 K
*V* = 885.2 (2) Å^3^	Block, colourless
*Z* = 4	0.45 × 0.23 × 0.18 mm
*F*(000) = 344	

### Data collection

**Table:**

Agilent Xcalibur Atlas Gemini diffractometer	2396 independent reflections
Radiation source: Enhance (Mo) X-ray Source	1348 reflections with *I* > 2σ(*I*)
Graphite monochromator	*R*_int_ = 0.038
Detector resolution: 10.5564 pixels mm^-1^	θ_max_ = 29.8°, θ_min_ = 3.3°
ω scans	*h* = −11→11
Absorption correction: analytical (CrysAlis PRO; Agilent, 2013)	*k* = −9→9
*T*_min_ = 0.896, *T*_max_ = 0.952	*l* = −20→20
15448 measured reflections	

### Refinement

**Table:**

Refinement on *F*^2^	Primary atom site location: structure-invariant direct methods
Least-squares matrix: full	Secondary atom site location: difference Fourier map
*R*[*F*^2^ > 2σ(*F*^2^)] = 0.047	Hydrogen site location: mixed
*wR*(*F*^2^) = 0.141	H atoms treated by a mixture of independent and constrained refinement
*S* = 1.02	*w* = 1/[σ^2^(*F*_o_^2^) + (0.0592*P*)^2^ + 0.086*P*] where *P* = (*F*_o_^2^ + 2*F*_c_^2^)/3
2396 reflections	(Δ/σ)_max_ < 0.001
114 parameters	Δρ_max_ = 0.11 e Å^−^^3^
0 restraints	Δρ_min_ = −0.15 e Å^−^^3^
0 constraints	

### Fractional atomic coordinates and isotropic or equivalent isotropic displacement parameters (Å^2^)

**Table:**

	*x*	*y*	*z*	*U*_iso_*/*U*_eq_	Occ. (<1)
N1	0.94381 (13)	0.15771 (14)	0.28174 (8)	0.0457 (3)	
O1	1.08946 (11)	0.15149 (14)	0.31104 (7)	0.0590 (3)	
C2	0.83146 (16)	0.15843 (17)	0.34397 (9)	0.0470 (3)	
H2	0.8559	0.1563	0.4065	0.056*	
C3	0.68056 (16)	0.16230 (18)	0.31562 (10)	0.0486 (4)	
C4	0.64802 (17)	0.16441 (19)	0.22220 (10)	0.0514 (4)	
H4	0.5469	0.1659	0.2018	0.062*	
C5	0.76390 (17)	0.16434 (19)	0.15834 (10)	0.0510 (4)	
C6	0.91205 (16)	0.16163 (18)	0.19077 (9)	0.0494 (4)	
H6	0.9916	0.1625	0.1492	0.059*	
C7	0.55650 (18)	0.1629 (2)	0.38570 (12)	0.0671 (5)	
H7A	0.4713	0.0891	0.3630	0.101*	0.5
H7B	0.5246	0.2929	0.3968	0.101*	0.5
H7C	0.5943	0.1071	0.4421	0.101*	0.5
H7D	0.5962	0.2119	0.4431	0.101*	0.5
H7E	0.5200	0.0341	0.3944	0.101*	0.5
H7F	0.4741	0.2432	0.3644	0.101*	0.5
C8	0.7304 (2)	0.1655 (3)	0.05650 (11)	0.0765 (5)	
H8A	0.6742	0.0517	0.0399	0.115*	0.5
H8B	0.8244	0.1682	0.0237	0.115*	0.5
H8C	0.6711	0.2773	0.0409	0.115*	0.5
H8D	0.7755	0.0543	0.0288	0.115*	0.5
H8E	0.7721	0.2799	0.0297	0.115*	0.5
H8F	0.6220	0.1630	0.0460	0.115*	0.5
O2	1.1840 (2)	−0.1483 (2)	0.43302 (9)	0.0959 (5)	
H2A	1.156 (3)	−0.057 (4)	0.3962 (19)	0.144*	
H2B	1.193 (3)	−0.253 (5)	0.401 (2)	0.144*	
O3	1.27722 (15)	0.01408 (19)	0.17111 (9)	0.0752 (4)	
H3A	1.229 (3)	0.038 (3)	0.2219 (17)	0.113*	
H3B	1.315 (3)	−0.101 (4)	0.1759 (16)	0.113*	

### Atomic displacement parameters (Å^2^)

**Table:**

	*U*^11^	*U*^22^	*U*^33^	*U*^12^	*U*^13^	*U*^23^
N1	0.0437 (7)	0.0402 (6)	0.0533 (7)	−0.0005 (5)	0.0029 (5)	0.0008 (5)
O1	0.0422 (6)	0.0642 (7)	0.0704 (7)	0.0006 (4)	−0.0036 (5)	0.0010 (5)
C2	0.0520 (9)	0.0417 (7)	0.0474 (7)	−0.0001 (6)	0.0055 (6)	0.0015 (6)
C3	0.0476 (8)	0.0413 (7)	0.0570 (8)	0.0003 (6)	0.0078 (6)	0.0042 (6)
C4	0.0438 (8)	0.0484 (8)	0.0620 (9)	0.0006 (6)	−0.0026 (6)	0.0028 (6)
C5	0.0573 (9)	0.0448 (7)	0.0509 (8)	0.0018 (6)	0.0008 (6)	0.0023 (6)
C6	0.0518 (9)	0.0465 (8)	0.0502 (8)	0.0015 (6)	0.0099 (6)	0.0007 (6)
C7	0.0547 (10)	0.0762 (10)	0.0711 (11)	0.0031 (8)	0.0165 (8)	0.0062 (8)
C8	0.0840 (13)	0.0906 (13)	0.0546 (10)	0.0056 (10)	−0.0031 (9)	0.0001 (8)
O2	0.1413 (14)	0.0874 (10)	0.0584 (8)	0.0096 (9)	−0.0175 (8)	−0.0027 (6)
O3	0.0737 (9)	0.0696 (8)	0.0828 (9)	0.0122 (6)	0.0162 (6)	0.0063 (6)

### Geometric parameters (Å, º)

**Table:**

N1—O1	1.3404 (14)	C7—H7C	0.9600
N1—C6	1.3463 (18)	C7—H7D	0.9600
N1—C2	1.3486 (17)	C7—H7E	0.9600
C2—C3	1.380 (2)	C7—H7F	0.9600
C2—H2	0.9300	C8—H8A	0.9600
C3—C4	1.382 (2)	C8—H8B	0.9600
C3—C7	1.503 (2)	C8—H8C	0.9600
C4—C5	1.388 (2)	C8—H8D	0.9600
C4—H4	0.9300	C8—H8E	0.9600
C5—C6	1.375 (2)	C8—H8F	0.9600
C5—C8	1.504 (2)	O2—H2A	0.87 (3)
C6—H6	0.9300	O2—H2B	0.87 (3)
C7—H7A	0.9600	O3—H3A	0.87 (3)
C7—H7B	0.9600	O3—H3B	0.87 (3)
			
O1—N1—C6	119.52 (11)	H7A—C7—H7C	109.5
O1—N1—C2	119.37 (11)	H7B—C7—H7C	109.5
C6—N1—C2	121.11 (12)	C3—C7—H7D	109.5
N1—C2—C3	120.53 (13)	C3—C7—H7E	109.5
N1—C2—H2	119.7	H7D—C7—H7E	109.5
C3—C2—H2	119.7	C3—C7—H7F	109.5
C2—C3—C4	118.32 (13)	H7D—C7—H7F	109.5
C2—C3—C7	119.98 (13)	H7E—C7—H7F	109.5
C4—C3—C7	121.70 (14)	C5—C8—H8A	109.5
C3—C4—C5	121.00 (14)	C5—C8—H8B	109.5
C3—C4—H4	119.5	H8A—C8—H8B	109.5
C5—C4—H4	119.5	C5—C8—H8C	109.5
C6—C5—C4	118.00 (13)	H8A—C8—H8C	109.5
C6—C5—C8	120.36 (14)	H8B—C8—H8C	109.5
C4—C5—C8	121.64 (15)	C5—C8—H8D	109.5
N1—C6—C5	121.03 (13)	C5—C8—H8E	109.5
N1—C6—H6	119.5	H8D—C8—H8E	109.5
C5—C6—H6	119.5	C5—C8—H8F	109.5
C3—C7—H7A	109.5	H8D—C8—H8F	109.5
C3—C7—H7B	109.5	H8E—C8—H8F	109.5
H7A—C7—H7B	109.5	H2A—O2—H2B	108 (3)
C3—C7—H7C	109.5	H3A—O3—H3B	107 (2)
			
O1—N1—C2—C3	−179.17 (10)	C3—C4—C5—C6	0.16 (19)
C6—N1—C2—C3	0.31 (18)	C3—C4—C5—C8	179.65 (13)
N1—C2—C3—C4	0.33 (18)	O1—N1—C6—C5	178.76 (11)
N1—C2—C3—C7	179.96 (12)	C2—N1—C6—C5	−0.73 (18)
C2—C3—C4—C5	−0.56 (19)	C4—C5—C6—N1	0.48 (19)
C7—C3—C4—C5	179.82 (12)	C8—C5—C6—N1	−179.01 (13)

### Hydrogen-bond geometry (Å, º)

**Table:**

*D*—H···*A*	*D*—H	H···*A*	*D*···*A*	*D*—H···*A*
O2—H2*A*···O1	0.87 (3)	1.98 (3)	2.8489 (18)	179 (3)
O2—H2*B*···O3^i^	0.87 (3)	1.94 (3)	2.815 (2)	178 (3)
O3—H3*A*···O1	0.87 (3)	1.96 (3)	2.8053 (17)	162 (2)
O3—H3*B*···O1^i^	0.87 (3)	1.92 (3)	2.7875 (17)	176 (2)
C4—H4···O3^ii^	0.93	2.62	3.484 (2)	155
C2—H2···O2^iii^	0.93	2.36	3.246 (2)	158
C7—H7*D*···O2^iii^	0.96	2.65	3.453 (2)	141

Symmetry codes: (i) −*x*+5/2, *y*−1/2, −*z*+1/2; (ii) *x*−1, *y*, *z*; (iii) −*x*+2, −*y*, −*z*+1.
